# Design and Motion Performance Analysis of Turbulent AUV Measuring Platform

**DOI:** 10.3390/s22020460

**Published:** 2022-01-08

**Authors:** Yunli Nie, Dalei Song, Zhenyu Wang, Yan Huang, Hua Yang

**Affiliations:** 1 College of Ocean Science and Engineering, Shandong University of Science and Technology, No. 579 Qianwangang Rd., Qingdao 266590, China; nieyunli@sdust.edu.cn; 2 College of Engineering, Ocean University of China, No. 238 Songling Rd., Qingdao 266100, China; 3 Institute for Advanced Ocean Study, Ocean University of China, No. 238 Songling Rd., Qingdao 266100, China; 4 Shenyang Institute of Automation Chinese Academy of Sciences, Shenyang 110016, China; wangzhenyu@sia.cn (Z.W.); huangy@sia.cn (Y.H.); 5 College of Information Science and Engineering, Ocean University of China, No. 238 Songling Rd., Qingdao 266100, China; hyang@ouc.edu.cn

**Keywords:** turbulence measurements, AUV, sensor integration, CFD, motion simulation, sea-trials

## Abstract

The use of a multi-functional autonomous underwater vehicle (AUV) as a platform for making turbulence measurements in the ocean is developed. The layout optimization of the turbulence package and platform motion performance are limitation problems in turbulent AUV design. In this study, the computational fluid dynamics (CFD) method has been used to determine the optimized layout position and distance of the shear probe integrated into an AUV. When placed 0.8 D ahead of the AUV nose along the axis, the shear probe is not influenced by flow distortion and can contact the water body first. To analyze the motion of the turbulence AUV, the dynamic model of turbulence AUV for planar flight is obtained. Then, the mathematical equations of speed and angle of attack under steady-state motion have also been obtained. By calculating the hydrodynamic coefficients of the turbulence AUV and given system parameters, the simulation analysis has been conducted. The simulation results demonstrated that the speed of turbulent AUV is 0.5–1 m/s, and the maximum angle of attack is less than 6.5°, which meets the observation requirements of the shear probe. In addition, turbulence AUV conducted a series of sea-trials in the northern South China Sea to illustrate the validity of the design and measurement. Two continuous profiles (1000 m) with a horizontal distance of 10 km were completed, and numerous high-quality spatiotemporal turbulence data were obtained. These profiles demonstrate the superior flight performance of turbulence AUV. Analysis shows that the measured data are of high quality, with the shear spectra being in very good agreement with the Nasmyth spectrum. Dissipation rates are consistent with background shear. When shear velocity is weak, the measurement of dissipation rate is 10^−10^ W Kg^−1^. All indications are that the turbulence AUV is suitable for long-term, contiguous ocean microstructure measurements, which will provide data needed to understand the temporal and spatial variability of the turbulent processes in the oceans.

## 1. Introduction

Ocean turbulence and the consequent dissipation of energy play an important role in the spread of contaminants, sedimentation processes, and biogeochemical fluxes within water masses, facilitating ocean–atmosphere gas exchange and global ocean circulation [1,2]. Turbulent mixing is also recognized as a key parameter in global climate models, used for understanding and predicting future climate change [3]. An understanding of the distribution of turbulent energy under various background conditions is therefore essential.

According to Lueck et al. [4], a wide variety of both vertical and horizontal turbulence instruments has been developed. For example, from 1970 to now, research groups have developed a variety of vertical profilers to meet specific scientific purposes. The vertical profiler is a very quiet platform without mechanical vibration. The use of these profilers demonstrated the spatial and temporal variability of turbulence structures [5]. However, the vertical profiler is limited to vertical profiles in one dimensional, one-time characterizations of the turbulent field and cannot provide a horizontal sampling [6]. More and more observations show that ocean turbulence is intermittent in time and space, and noncontiguous in regimes of strong stratification [7]. To resolve this structure, researchers began to measure turbulence with horizontally profiling instruments, including towed bodies, submersibles, and moored platforms. Early horizontal turbulence instruments were limited by their cost for deployment and were not widely used. Recently, several small AUVs with a length of 2–4 m equipped with shear probes have been developed and used to measure ocean turbulence. These include the Ocean Explorer, REMUS AUV, and Glider, etc. [8,9,10,11,12], whose dissipation rates are as low as 10^−10^ W kg^−1^. The AUV has flexible mission types, which can realize horizontal, vertical, as well as bottom and surface observation tasks. Thus, using AUV as a turbulence measurement platform can realize long-duration, continuous measurement, which will provide data needed to understand the temporal and spatial variability of the turbulent processes and support the examination of theories of turbulent cascade and stationarity in the oceans.

Despite the advantages of AUV-based turbulence measurements, there are still great technical challenges in integrating turbulence sensors on AUV. The most common turbulence sensor is the airfoil shear probe, which uses the potential flow theory to measure the cross-stream component of velocity perpendicular to the direction of travel [13]. Flow distortion occurs when the free streamflow approaches the AUV body, which will change the nearby vorticity and velocity field. Consequently, shear probe measurements require to be far away from the flow distortion region caused by the AUV. This is the first technical challenge in the design of the implementation shear probe on an AUV. Wyngaard et al. [14] use the Taylor series expansion technique to assess errors associated with turbulence measurements ahead of an axisymmetric body. Using the ellipsoid model with an aspect ratio of 5:1 (L/D, where L is the length of the body and D is the maximum diameter), they found that the fractional errors in turbulent velocity statistics are on the order of 10% when measured along the axis on the plane of 0.5 D ahead of the body. The errors generally decrease with the increase of distance. According to this study, the researchers discussed the flow deformation of different platforms and installed the shear probe above and forward of the bow of the platform where flow distortion is small [8,15,16]. However, these studies only used empirical methods for analysis and did not give a detailed hydrodynamic estimation of the platform in the design.

The second limitation of AUV-based turbulence measurements is motion performance, including the angle of attack (AOA or α) and the speed (*U*), which are very sensitive to microstructure shear estimation. The shear probes output is a voltage proportional to the instantaneous cross-stream component (*u*) of the velocity field [17].

(1) $$E=2\sqrt{2}S\rho Uu$$

where S is the sensitivity of the shear probe, ρ is the in situ density, and *U* is the flow past the sensor which, in this application, is the AUV speed through the water. This equation is valid when the AOA is within ±20° relative to the oncoming flow. Outside of this range, the *u* will be mixed with the downstream velocity fluctuations and the potential flow theory of the probes no longer applies. The *U* is an important parameter in the processing of microstructure shear, which is related to the properties of the shear probe and the time scale of dissipating eddies, usually need greater than 0.3 m/s [18]. Using Taylor’s frozen turbulence hypothesis [19], we can convert time series E(t) to a space series, whereby

(2) $$\frac{\partial u}{\partial x}=\frac{1}{U}\frac{\partial u}{\partial t}=\frac{1}{2\sqrt{2}S\rho }\frac{1}{U^{2}}\frac{\partial E}{\partial t}$$

For isotropic turbulence, the turbulent dissipation rate ε calculated as

(3) $$\varepsilon =\frac{15}{2}v\bar{{\left(\frac{\partial u}{\partial x}\right)}^{2}}\propto \frac{1}{U^{4}}\bar{{\left(\frac{\partial E}{\partial t}\right)}^{2}}$$

where x represents the distance in the AUV path direction, v is the kinematic viscosity, and the bar denotes a mean. According to Equation (3), the ε scales with *U*^4^, so the small errors of *U* will introduce a significant bias to the ε. For AUV-based turbulence measurements, AOA is not measured and uncommonly the AUV is equipped with a device to directly measure the speed through water, so these values must be computed from the dynamic model. The stable and accurate motion performance of the AUV is very important for turbulence observation. 

In this paper, a long-rang and multi-motion mode AUV was developed and used as the platform for turbulence measurement. To ensure that the shear probe can be measured in undisturbed water and minimize its effect on the AUV’s flight characteristics, the hydrodynamic parameters were estimated using the CFD method and its integrated layout design on AUV was discussed. Because the shear probe is very sensitive to the AUV speed and AOA, the dynamic model of the turbulence AUV was developed by using an analytical method based on Newton–Euler and its motion performance was simulated. Then, sea trials were carried out. The self-developed AUV and the turbulence package are described in Section 2. Section 3 estimates the hydrodynamic parameters of the turbulence AUV under different states and determines the layout position. Section 4 describes the AUVs dynamic model and the simulation results of its motion performance in the longitudinal plane. Section 5 presents and discusses the sea trial results, including the flight performance of the turbulence AUV and the microstructure shear data analysis. Section 6 contains the summary and conclusions.

## 2. Turbulence AUV

**Figure 1:**
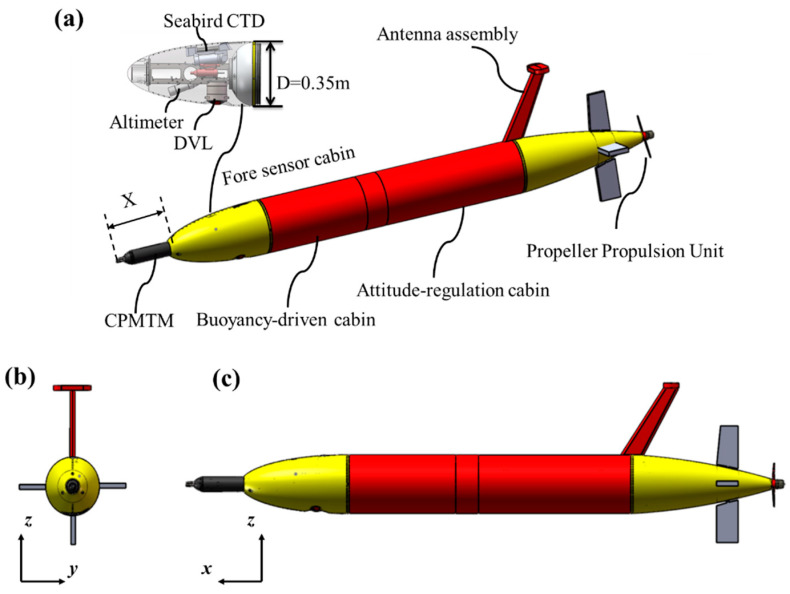
The turbulence AUV configuration. (**a**) Detailed structural components and oceanographic sensors of turbulent AUV. The CPMTM is housed in the center of the fore sensor cabin with the shear probes ahead of the nose by X distance. (**b**) Front view. (**c**) Side view.

The long-range turbulence AUV platform is shown in Figure 1. The vehicle adopts a modular design idea, which consists of a conical fore sensor cabin, buoyancy-driven cabin, attitude-regulation cabin, controller module, power module, propulsion system, and antenna assembly, etc. [20]. It is 3 m long (L) and 0.35 m in diameter (D), with a mass of 200 kg. The entire system is powered by battery packs, can dive to 2000 m underwater for ocean surveys, and has a 1500 km endurance. To meet different observation tasks, the turbulence AUV is designed to combine the characteristics of the buoyancy-driven glider and the conventional AUV. Therefore, it has a variety of flexible motion modes, such as depth-following mode, yo-yo mode like a glider, and the mode combining depth-following and yo-yo mode. 

The turbulence AUV onboard sensors include a 1 MHz Doppler velocity log (DVL), a Seabird conductivity, temperature, depth (CTD) data logger (with dissolved oxygen), an altimeter, and a cross-platform instrument for microstructure turbulence measurements (CPMTM). The DVL is installed downward-looking designed to continuously measure bottom-track velocities and three-dimensional current profiles. The turbulence measurements are made concomitantly with high spatial resolution measurements of CTD. This set of sensors on turbulence AUV allows for quantification of the key dynamical and kinematical turbulent and fine-scale physical processes. All sensors are mounted in the fore sensor cabin located at the bow (Figure 1). This layout allows sensors to be located away from the aft propulsion system, improving data sampling accuracy while preserving the AUV’s low drag profile. 

**Figure 2:**
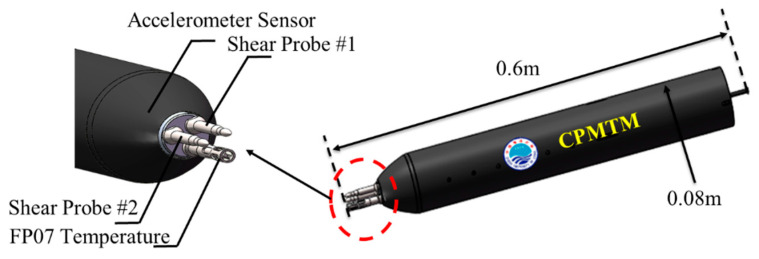
The schematic of the CPMTM, and an array of turbulence sensors in the nose.

The CPMTM is designed by the Ocean University of China [21], as shown in Figure 2. It is housed in a pressure case of 0.08 m diameter and is approximately 0.6 m long. It is equipped with two airfoil shear probes, a fast thermistor, and a 3-axis accelerometer. The shear probes are mounted orthogonally to measure cross-stream velocity fluctuations ∂y⁄∂x and vertical velocity gradient ∂z⁄∂x. The fast thermistor to measure microstructure temperature and its fluctuation and the 3-axis accelerometer to measure the level of vibration during observation. The CPMTM is powered by the AUV’s battery. The sampling rate is 1024 Hz on all turbulence channels (shear and temperature) and 512 Hz for the accelerometer channel. The CPMTM is mounted to the center of the AUV fore sensor cabin (Figure 1b,c) with the same coordinate system. The shear probe is located at the X distance from the nose of the AUV, outside the region of flow deformation (Figure 1a), and the optimal layout distance will be described in Section 3.

## 3. The Layout Optimization of CPMTM

According to the principle of the shear probe, its measurement must be in the undisturbed area to avoid measurement error. However, if the layout distance between the shear probe and the AUV nose is too large, it will affect the motion performance of AUV. To determine the optimal layout distance, the CFD method was used to estimate the hydrodynamic properties of the turbulence AUV in various angles of attack and speeds. 

**Figure 3:**
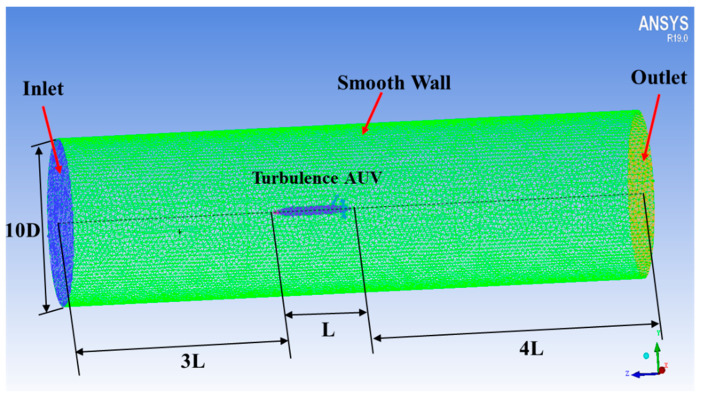
The computational domain and unstructured tetrahedron grid of the turbulence AUV. L is the length of the turbulence AUV and D is the diameter. The computational domain is set to be cylindrical with a diameter of 10 D, the inlet boundary is located at the 3 L head of the turbulence AUV, and the pressure outlet condition is installed 4 L downstream.

**Figure 4:**
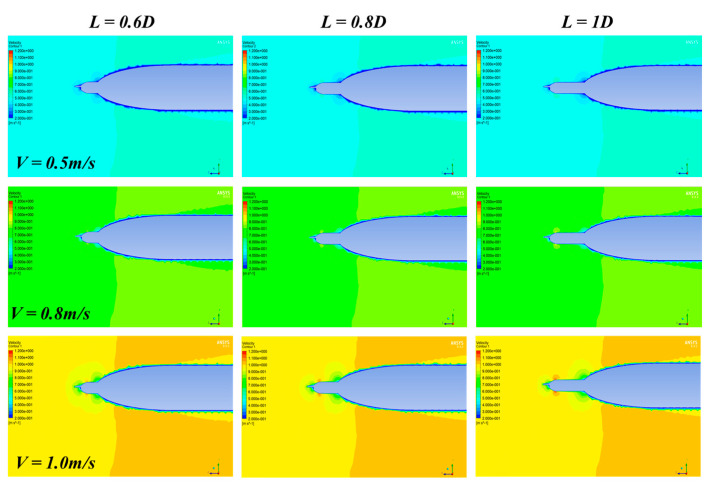
Velocity vectors of the turbulence AUV under various models with 0° AOA. The rows represent the same velocity of the flow field and the columns represent the same distance X.

**Figure 5:**
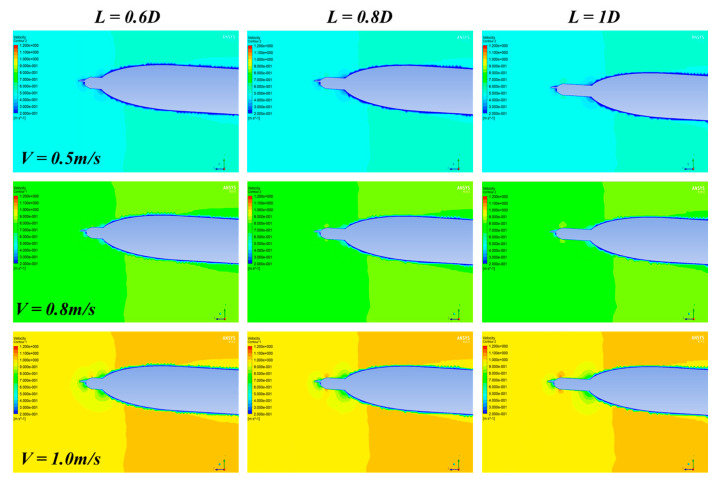
Velocity vectors of the turbulence AUV under various models with 3° AOA. The rows represent the same velocity of the flow field and the columns represent the same distance X.

**Figure 6:**
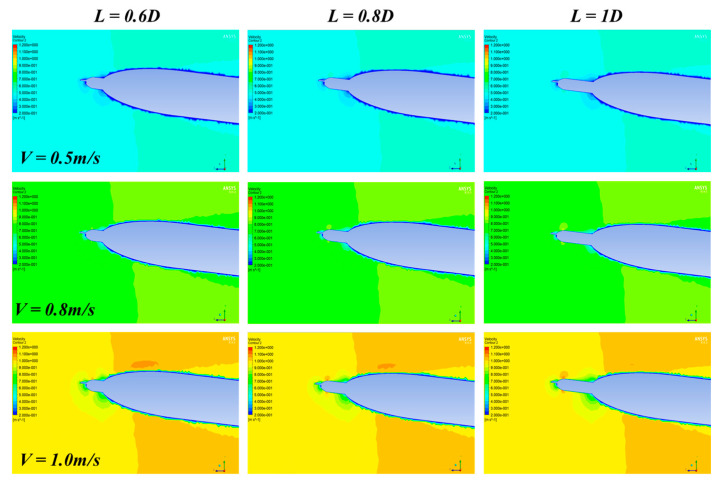
Velocity vectors of the turbulence AUV under various models with 6° AOA.

The Ansys and Fluent software are used for CFD analysis. The turbulence AUV model used for simulation is the same as the real model (Figure 1). The flow field is set to be cylindrical with a diameter of 10 D, the inlet boundary is located at the 3 L head of the turbulence AUV, and the pressure outlet is located at 4 L downstream, as shown in Figure 3. A no-slip condition is forced at the AUV wall, and at the surrounding area, far-field free-slip wall conditions are applied. The CFD analysis is based on unstructured tetrahedron cells as the meshing grid. To improve the accuracy of CFD simulation, the grid of CPMTM and AUV nose is locally encrypted. The velocities of the flow field were used for the analysis using 0.5 m/s, 0.8 m/s, and 1.0 m/s (the maximum design speed of AUV is 1.0m/s) respectively. When the AOA sign is opposite, the flow field is approximately symmetrical. Therefore, the flow field analysis is carried out under 0°, 3°, and 6° AOA. Distance X is set to 0.6 D, 0.8 D, 1 D. Figure 4, Figure 5 and Figure 6 show the snapshot of the velocity vectors for the turbulence AUV in different simulation conditions.

It can be seen a low-velocity region is formed around the head of AUV (Figure 4). Those low-velocity regions can bond if the distance between CPMTM and AUV is too short (Figure 4a,d,g). With the increase of inlet velocity, the area of the low-velocity region gradually increases and presents a certain linear relationship. This indicates that the larger of AUV velocity, the larger flow distortion area caused by the body, the distance should be larger between CPMTM and AUV. When the distance between CPMTM and AUV is X = 0.6 D, the low-velocity region formed by the AUV completely wraps the CPMTM. The flow field around the CPMTM has been distorted, which cannot reflect the real flow field information, resulting in an inaccurate measurement. When X = 0.8 D, the low-velocity region is completely separated from the CPMTM. At this time, the CPMTM is not influenced by flow distortion and can contact the water body first (Figure 4b,e,h). The CPMTM has also gotten rid of the influence of flow distortion on the condition that distance is 1 D (Figure 4c,f,i).

Figure 5 and Figure 6 show the simulation results of the flow field at 3° and 6° AOA respectively. The results show that when there is an angle of attack, the distribution of velocity field around the axis of turbulence AUV is no longer symmetrical. With the increase of the AOA, the area of the low-velocity region above the AUV body decreases gradually, and the area below the AUV body increases gradually. Compared with the 0° AOA, the variation tendency is basically consistent. The flow field disturbance caused by the AUV can also be avoided when the distance is 0.8 D. Hence, the layout distance of CPMTM is at least 0.8 D to extend the head of AUV.

**Figure 7:**
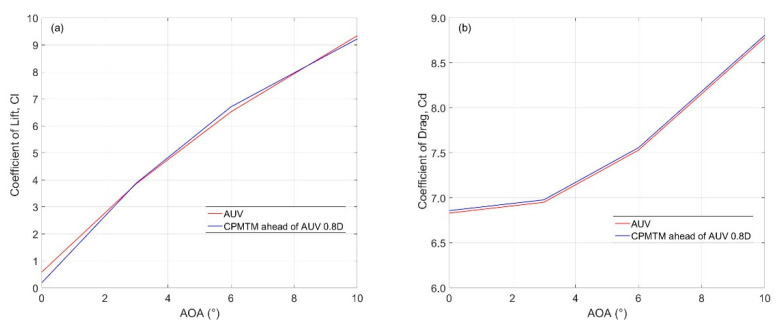
Lift (**a**) and drag force (**b**) coefficient over the angle of attack of the AUV before and after integrating CPMTM.

The rows represent the same velocity of the flow field and the columns represent the same distance X.The integration of CPMTM into AUV will cause the hydrodynamic variation of AUV, which may affect the navigation economy and stability of AUV. Therefore, the hydrodynamics variation before and after integrating CPMTM should be as small as possible. In the CFD analysis, lift force (C_L_) and drag force (C_D_) coefficients were calculated at various angles of attack and velocities. The results of AOA between 0° and 10°, AUV economic speed of 0.5 m/s, and layout distance X = 0.8 D were used for the analysis. Figure 7a shows the lift force coefficient of AUV and turbulence AUV over the AOA. The graph shows that the lift force coefficient increased when the AOA increased, the lift coefficient variation little when the AUV integrates CPMTM. The drag force coefficient over the AOA is shown in Figure 7b. The figure shows that the drag coefficient increased when the AOA increased, and when AUV integrated CPMTM the drag coefficient increased slightly (blue line). Thus, the optimal layout distance that CPMTM bulges over the head of AUV is X = 0.8 D, which is far away from the flow distortion caused by the AUV nose and ensures that the hydrodynamic variations before and after the AUV integration with CPMTM are very small.

## 4. Motion Performance Analysis of the Turbulence AUV

### 4.1. Dynamic Model of Turbulence AUV for Planar Flight

**Figure 8:**
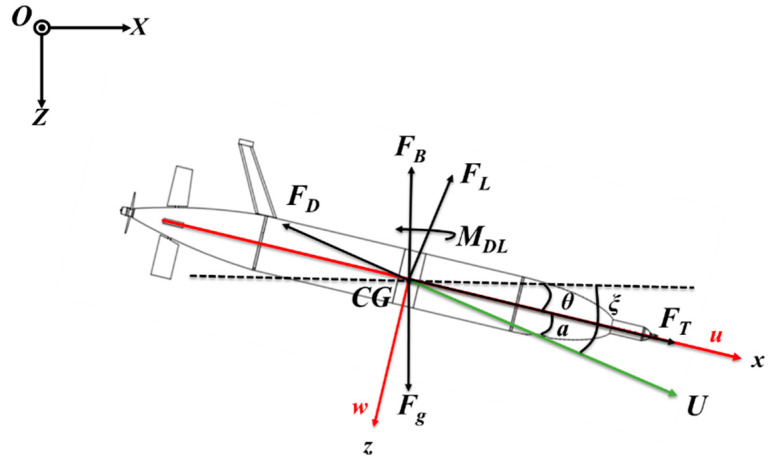
The force analysis of turbulence AUV in the vertical plane (descent) and definition of coordinate systems. The forces working on the turbulence AUV are buoyancy *F_B_*, gravity *F_g_*, lift *F_L_*, drag *F_D_*, thrust *F_T_*, and moment *MDL*. *θ* is the pitch angle, α is the angle of attack, 𝜉 is the path angle. *u* and *w* are the horizontal and vertical turbulence AUV speed components in a georeferenced coordinate system, respectively. The earth-based coordinates are X, Y (both horizontal), and Z (downward), while the AUV-based coordinates are x (along axis), y (to port), and z.

The second key aim of this paper is to establish the turbulence AUV dynamic model and obtain the motion parameters to determine that the U is greater than 0.3 m/s and the AOA is within ±20°. The dynamic model of the turbulence AUV was formulated by using an analytical method based on Newton–Euler. The definition of the coordinate system is shown in Figure 8. The earth-based coordinate is O-XYZ and the origin is fixed at the sea surface. In the AUV-based coordinates (x, y, z), the x axis is directed forward along the main central axis of the AUV, in its direction of motion. The y axis points to the port side of the instrument, and the z axis is nominally downward.

The yo-yo motion in the longitudinal plane is the main movement of the turbulence AUV, and this motion mode can be deemed to be quasi-steady flight. Therefore, the simplified dynamic model of turbulence AUV in the X–Z plane is used to calculate its motion parameters. The forces act on the turbulence AUV are buoyancy *F_B_*, gravity *F_g_*, lift *F_L_*, drag *F_D_*, and thrust *F_T_*, as shown in Figure 8. For the sake of clarity, all forces are schematized to originate from the center of gravity (*CG*). The steady force and moment are balanced, as follows:

(4) $$F_{B}-F_{g}+F_{D}\sin\left(\xi \right)+F_{L}\cos\left(\xi \right)-F_{T}\sin\left(\theta \right)=0$$

(5) $$F_{D}\cos\left(\xi \right)-F_{L}\sin\left(\xi \right)-F_{T}\cos\left(\theta \right)=0$$

where θ is the pitch angle and 𝜉 is the path angle, which is the sum of θ and *α*. The gravity *F_g_*, buoyancy *F_B_*, drag *F_D_*, lift *F_L_*, and thrust *F_T_* are given by:

(6) $$F_{g}=m_{g}g$$

(7) $$F_{B}=g\rho \left\{V_{g}\left[1-\varepsilon_{c}P+\alpha_{T}\left(T-T_{0}\right)\right]+\Delta V_{bp}\right\}$$

(8) $$F_{D}=\frac{1}{2}\rho SU^{2}\left(C_{D0}+\alpha^{2}C_{D1}\right)$$

(9) $$F_{L}=\frac{1}{2}\rho SU^{2}C_{L}\left(\alpha \right)$$

(10) $$F_{T}=K_{T}\rho n^{2}d_{p}^{4}$$

In Equations (6)–(10), $m_{g}$ is the mass of the turbulence AUV and g is the acceleration due to gravity, ρ is the in situ density, $V_{g}$ is the turbulence AUV volume at atmospheric pressure, $\varepsilon_{c}$ is the coefficient of compressibility, P is the water pressure, $\alpha_{T}$ is the thermal expansion coefficient, *T* is the water temperature, $T_{0}$ is a reference temperature, $\Delta V_{bp}$ is the buoyancy change achieved by the buoyancy-driven system of the AUV, S is the total surface area of the wings, $C_{D0}$ and $C_{D1}$ are the parasite and induced drag coefficient, respectively, $C_{L}\left(\alpha \right)$ is linear in the angle of attack,$\;C_{L}\left(\alpha \right)=a\alpha$, $K_{T}$ is the propeller thrust coefficient, n is the propeller speed (rpm, revolutions per minute), and $d_{p}$ is the diameter of propeller (m).

Substituting Equations (6)–(10) into Equations (4) and (5) respectively, the expression of *U* can be calculated by either eliminating $F_{D}$ or $F_{L}$ from (4) and (5).

(11) $$U=\sqrt{\frac{\Delta B+F_{T}\sin\left(\theta \right)}{cos\left(\theta +\alpha \right)C_{L}\left(\alpha \right)+\sin\left(\theta +\alpha \right)\left(C_{D0}+\alpha^{2}C_{D1}\right)}}$$

In addition, an expression for α is found by combining (5), and (8)–(10), yielding

(12) $$\alpha =\frac{C_{DO}+\alpha^{2}C_{D1}}{\mathrm{atan}\left(\theta +\alpha \right)}-\frac{HF_{T}\cos\left(\theta \right)}{\mathrm{asin}\left(\theta +\alpha \right)}\;$$

where ΔB is net buoyancy, $\Delta B=F_{B}-F_{g}$, and H is the calculated coefficient.

Equations (11) and (12) provide a model that can be used to calculate the steady-state motion parameters at any time by giving a range of values of net buoyancy (ΔB), pitch angle (*θ*), and in situ density, as well as a set of model coefficients of drag, lift, compressibility, and thermal expansion.

### 4.2. Simulations

The motion performance of turbulent AUV was simulated using the fourth-order Runge–Kutta method in MATLAB. Considering the economic travel of turbulent AUV and specific sampling requirement, the pitch angle is set within the range of ±15°. The maximum net buoyancy that can be provided is ±6 N, while the propeller speed range is 80–200 rpm, providing thrust up to 10 N. In the descent, we defined the pitch angle as negative and the net buoyancy as positive. To avoid singularity, the initial motion state *u* = 0.01 m/s when the turbulent AUV is at the surface. The hydrodynamic coefficients of turbulence AUV have been calculated based on the CFD simulation.

**Figure 9:**
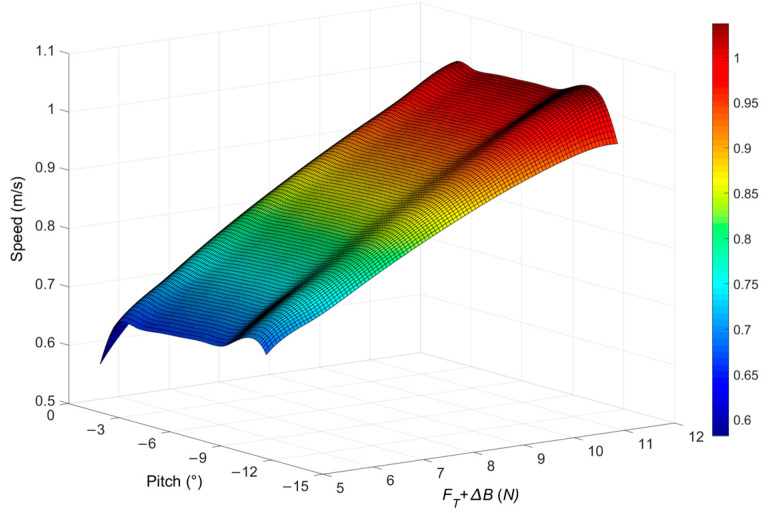
Speed simulation results of turbulence AUV.

- Speed;

Through the above parameters, we can obtain the relationship between the speed of the turbulent AUV (*U*) and net buoyancy (∆B), propeller thrust (*F_T_*), and pitch angle(*θ*), as shown in Figure 9. According to the simulation results, the speed of the turbulent AUV is approximately proportional to the net buoyancy and propeller thrust, that is, the speed increases with the increase of net buoyancy and propeller thrust. The speed increases with the increase of pitch angle. The speed of the turbulent AUV is 0.5~1.0 m/s, which meets the requirement of being greater than 0.3 m/s.

**Figure 10:**
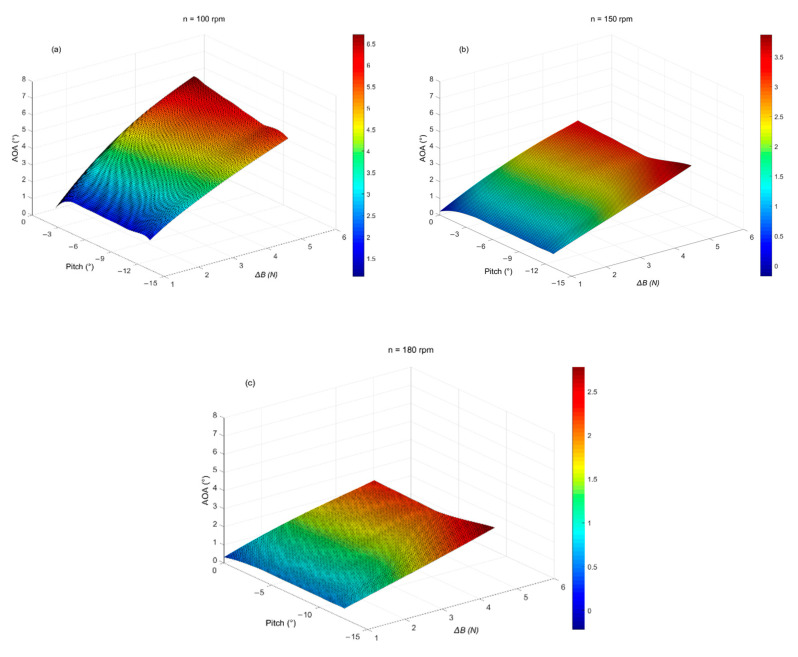
Relationship among AOA, net buoyance (∆*B*), and a pitch angle of turbulence AUV under different propeller speeds. (**a**) n = 100 rpm, (**b**) n = 150 rpm, (**c**) n = 180 rpm.

- AOA;

Figure 10 presents the simulation results of the AOA under different propeller speeds (n = 100 rpm, 150 rpm, 180 rpm) of turbulent AUV. According to Figure 10, when the pitch angle is constant, the AOA increases with the increase of net buoyancy, and the maximum value is 6.5° (Figure 10a). When the net buoyancy is constant, the AOA is smaller with the increase of pitch angle. However, as the propeller speed increases, the AOA decreases significantly. The maximum AOA is 3.7° at n = 150 rpm (Figure 10b) and 2.7° at n = 180 rpm (Figure 10c). Therefore, under the conditions of different pitch angles, net buoyancy, and propeller speeds, the corresponding AOA is less than 10°, which meets the constraint requirements of turbulence observation.

**Figure 11:**
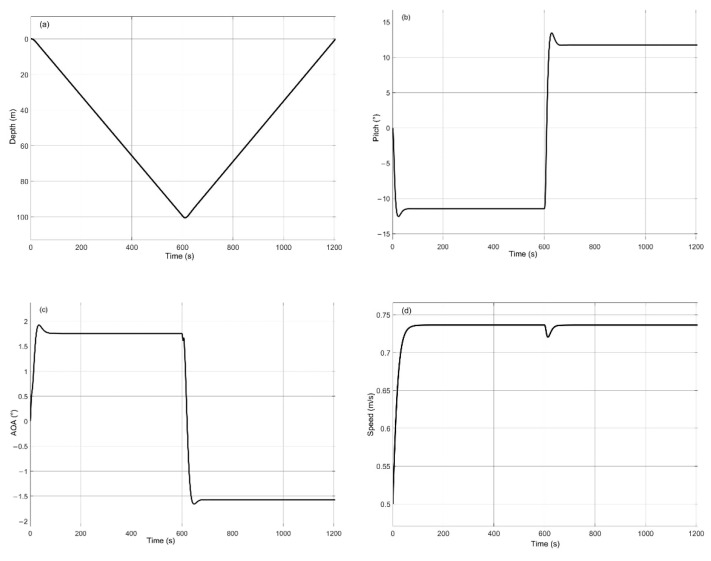
When $F_{T}$ = 6 N, ΔB = 1N, *θ* = 12° are set in yo-yo profile, the simulation results: (**a**) depth vs. time; (**b**) pitch vs. time; (**c**) AOA vs. time; (**d**) speed vs. time.

In detail, we set $F_{T}$ = 6 N, ΔB = 1 N, and *θ* = 12° and carried out simulation analysis on motion depth, pitch angle, angle of attack, and running speed in the yo-yo profile. The results are shown in Figure 11. According to Figure 11a, within the simulation time of the 1200 s, the maximum running depth of turbulent AUV is 100 m, and the corresponding horizontal sailing distance is about 853 m. The steady speed of the turbulent AUV is 0.73 m/s (Figure 11d). In Figure 11b,c, the pitch angle is −11.5° and the AOA is 1.7° in the descent. The pitch angle is 11.7° and the AOA is 1.6° in the ascend. 

## 5. Field Experiment

**Figure 12:**
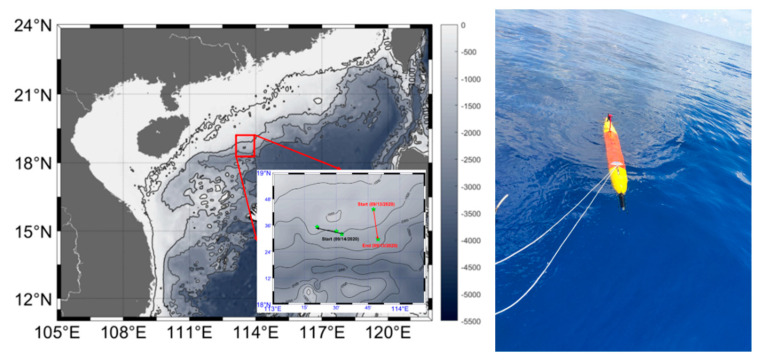
Bathymetry contours (in meters) of the northern South China Sea. The red rectangle indicates the location of the experiment. The enlarged view shows the track (red lines) of the turbulence AUV on 13 September 2020. The right picture is retrieved turbulence AUV from research vessel.

To test the motion performance and turbulence observation capabilities of the turbulence AUV, a field experiment was conducted on the slope of the northern South China Sea between 10 and 17 September 2020. The location of the experiment is shown in Figure 12, and the detailed track of the turbulence AUV is shown in the bottom right corner. The turbulence AUV was observed from north to south, starting at 08:20 on 13 September 2020 (local time), and ending at 16:18 on 13 September 2020. The turbulence AUV operated in a 13° yo-yo mode and depth-following mode. The depth ranged from the surface to 1000 m. The track consisted of 2 continuous profiles with the horizontal distance between profiles being on average 5 km apart.

### 5.1. Flight Performance of Turbulence AUV

**Figure 13:**
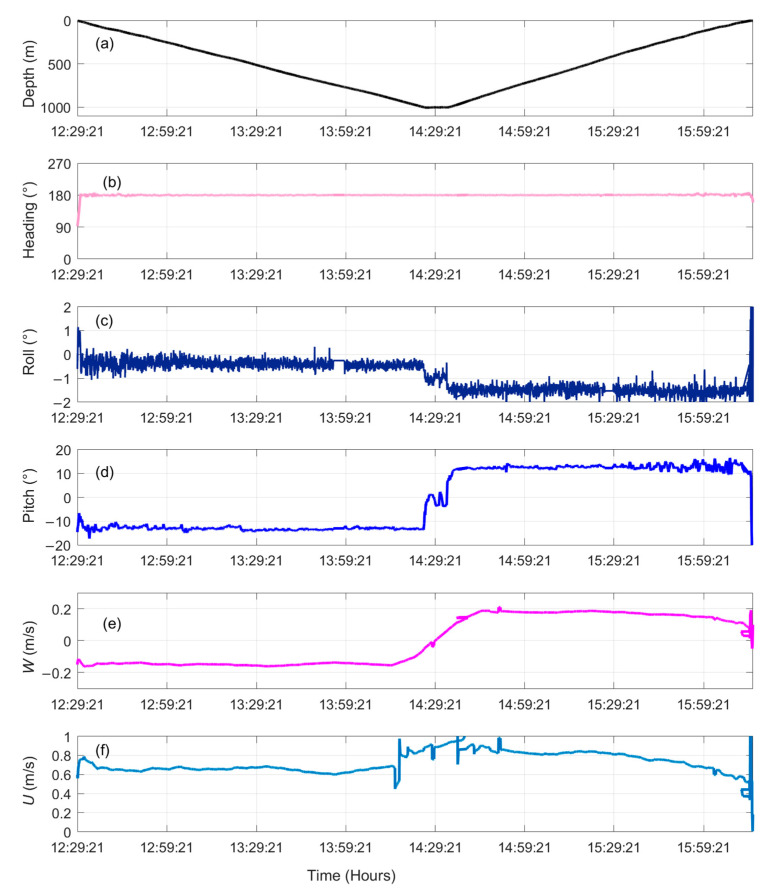
Time series of the turbulence AUV flight performance on 13 September 2020: (**a**) depth, (**b**) heading, (**c**) roll, (**d**) pitch, (**e**) vertical speed (*W*), and (**f**) speed (*U*).

The two profiles all gave similar results. Time series of flight performance data for one profile (from 12:29 to 16:18) are shown in Figure 13. The flight path of this profile was a straight line heading from north to south (Figure 13b), where the turbulence AUV performed a combined mode of yo-yo and depth-following (approximately 5 min) between surface and 1000 m depth. The roll was generally more variable but small, with typical amplitudes of 1° (Figure 13c). The pitch angles were approximately −13.12° and 12.65° during dive and climb, respectively (Figure 13d). The average speed along the turbulence AUV path was 0.65 m/s and 0.78 m/s during dive and climb, respectively, and 0.82 m/s in the depth-following (Figure 13f). Table 1 summarizes the typical speeds and pitch angles observed along the path. 

### 5.2. Shear Data Analysis

**Figure 14:**
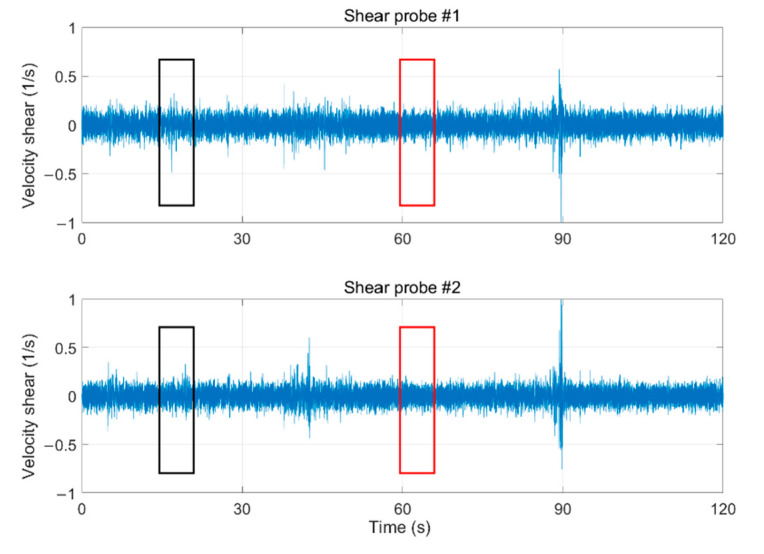
Filtered time series of velocity shear measured by turbulent AUV. The black and red rectangles denote the strong and weak velocity shear region respectively.

A segment of time series for the velocity shear (shear probes #1 and #2), 120 s long and collected during a steady descent, is shown in Figure 14. The velocity shear signals were bandpass filtered at 0.15 to 100 Hz to effectively remove low-frequency motions and high-frequency vibration signatures of the turbulence AUV, but this removal of signal content does not affect the calculation of the dissipation rate. According to the error processing criterion (Rayda’s criterion), if the measurement error is three times larger than the standard error, the singular data in the velocity shear signal have been deleted and substituted with the arithmetic average value. The amplitudes of velocity shear for both shear probe #1 and shear probe #2 vary from−0.5 to 0.5 s^−1^ and are fairly uniform.

**Figure 15:**
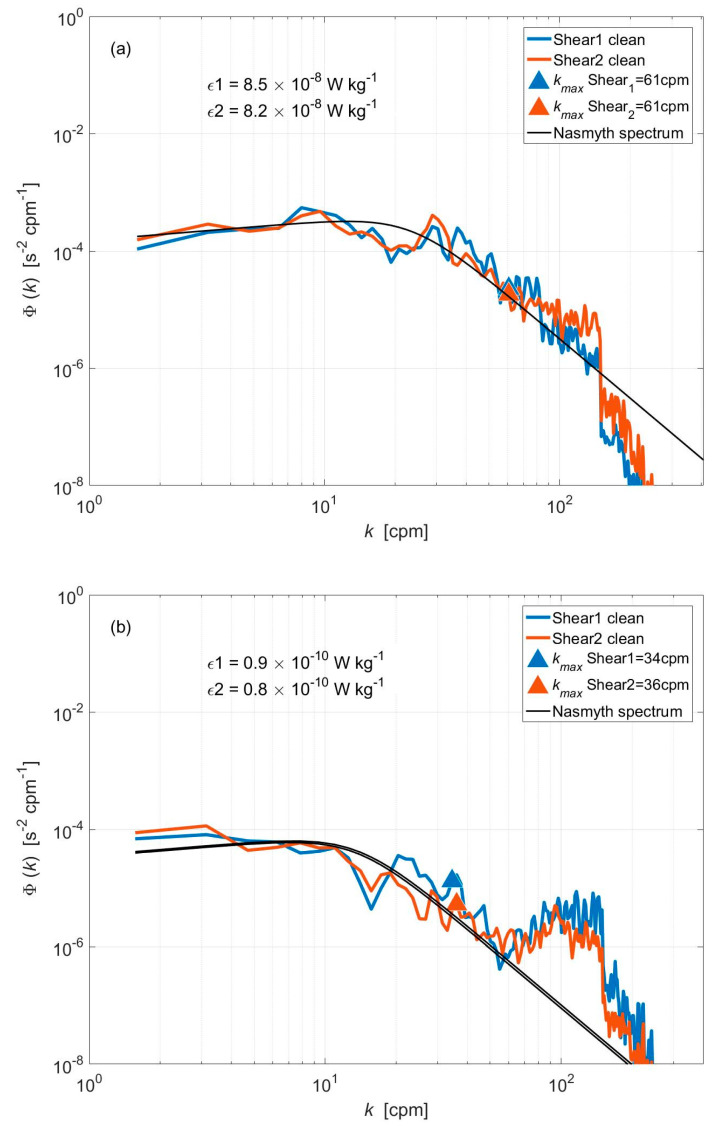
Shear spectra computed from the regions identified by the black (**a**) and red (**b**) rectangles in Figure 14. The green and orange lines represent the clean shear spectrum, and the black lines represent their corresponding Nasmyth spectra. The triangles (green, red) denote the upper limit of the integration wavenumber used in the computation of the dissipation rates. The corresponding ε estimate are included in each plot.

The frequency spectra for velocity shear are computed using Welch’s averaged periodogram method with a fast Fourier transform window length of 4 s [22,23]. Frequency spectra are turned into shear spectra by multiplying them by the average speed (*U*). To minimize contamination from vehicular motions and vibrations, we removed acceleration-coherent noise from the shear signal with the algorithm developed specifically for AUV by Goodman et al. [9]. The clean shear spectra ($\Phi_{u_{i}}\left(k\right),i=1,2)$ computed from the velocity shear signal in the rectangles of Figure 14 are shown in Figure 15 along with the corresponding scaled Nasmyth spectrum [24]. At two different times, the measured shear spectra of both shear probes agree with each other. In addition, they agree well with the corresponding Nasmyth spectrum (black line). Figure 15a shows the shear spectrum of the strong velocity shear region (black rectangle), where${\;\Phi }_{u_{1}}\left(k\right)$ (shear probe #1) and $\Phi_{u_{2}}\left(k\right)$ (shear probe #2) agree well with the Nasmyth spectrum between 2 and 61 cpm (cycles per meter). The shear spectrum calculated in the weak velocity shear region (red rectangle) is shown in Figure 15b. Both shear spectra agree well with the Nasmyth spectrum in the range of 2~36 cpm.

With the assumption of isotropic turbulence [5,19], the dissipation rate of turbulent kinetic energy (ε) is calculated by integrating the shear spectra as [21,25]

(13) $$\varepsilon_{i}=\frac{15}{2}v\bar{{\left(\frac{\partial u_{i}}{\partial x}\right)}^{2}}=\frac{15}{2}v\int_{k_{min}}^{k_{max}}\Phi_{u_{i}}\left(k\right)dk$$

where the lower integration limit$\;k_{min}$ and the upper limit of integration $k_{max}$ represent the wavenumber interval with good agreement between shear spectra and Nasmyth spectrum. The dissipation rates based on the measured shear spectra (Figure 15a) are ε1 = 8.5 × 10^−8^ W kg^−1^ and ε2 = 8.2 × 10^−8^ W kg^−1^. Meanwhile, in the weak velocity shear region (Figure 15b), the dissipation rates are a little lower, at ε1 = 0.9 × 10^−10^ W kg^−1^ and ε2 = 0.8 × 10^−10^ W kg^−1^. This shows that the turbulence AUV has extraordinarily low noise levels and can accurately measure ε as low as 10^−10^ W kg^−1^.

## 6. Conclusions

As a mobile autonomous ocean observation platform, AUV has the advantage of long endurance and large range, being unaffected by sea conditions. In this study, a long-range and multi-motion mode turbulence AUV was developed and used for turbulence measurement in the northern South China Sea. The presented work and field tests demonstrate the successful integration of the CPMTM into the AUV. In the turbulence AUV design, two key technical challenges were accomplished. Firstly, to avoid measurement errors of CPMTM caused by the flow distortion of the AUV body and to reduce the influence of motion performance of AUV after integrating CPMTM, the ideal layout positions are analyzed under different states with the method of CFD, and the distance of CPMTM bulges over the head of AUV is designed to be 0.8 D. Secondly, the dynamic model of turbulent AUV in the vertical plane was established and the motion performance was simulated. The simulation results show that the speed of turbulent AUV is 0.5–1 m/s, and the maximum angle of attack is less than 6.5°, which meets the observation requirements of the shear probe. During the field experiment, turbulence AUV operated in a combined mode of yo-yo and depth-following between the surface and 1000 m depth. Data were collected for approximately 8 h. Two profiles illustrate the stable flight performance of the turbulence AUV. The measured shear spectra fit well with the Nasmyth spectrum, and the dissipation rate was as low as 0.8 × 10^−10^ W kg^−1^, which suggests that the developed turbulence AUV is suitable for ocean microstructure measurements.

## Figures and Tables

**Table 1:** Typical average and standard deviation of pitch angle (*θ*), vertical speed (*W*)*,* and speed along the turbulence AUV path (*U*).

	*θ* (°)	*W* (m/s)	*U* (m/s)
Dive	−13.12 ± 0.66	−0.15 ± 0.01	0.65 ± 0.03
Climb	12.65 ± 0.95	0.17 ± 0.02	0.78 ± 0.09

## Data Availability

The data supporting this study are provided within this paper.
